# Versión española del *Nordic Musculoskeletal Questionnaire*: adaptación transcultural y validación en personal auxiliar de enfermería

**DOI:** 10.23938/ASSN.1066

**Published:** 2024-04-11

**Authors:** Laura Mateos-González, Julio Rodríguez-Suárez, Jose Antonio Llosa, Esteban Agulló-Tomás

**Affiliations:** 1 Universidad de Oviedo Departamento de Cirugía y Especialidades Médico-quirúrgicas Área de Fisioterapia Oviedo España; 2 Universidad de Oviedo Departamento de Psicología Oviedo España; 3 Universidad de Oviedo Escuela Universitaria Padre Ossó Departamento de Educación Social Oviedo España

**Keywords:** Encuestas y cuestionarios, Enfermedades musculoesqueléticas, Factores de riesgo, Salud laboral, Asistentes de enfermería, Surveys and questionnaires, Musculoskeletal diseases, Risk factors, Occupational health, Nursing assistants

## Abstract

**Fundamento:**

El objetivo de este trabajo es traducir, adaptar culturalmente y validar una versión española del *Nordic musculo- skeletal Questionnaire* (NMQ) en una muestra de personal auxiliar de enfermería.

**Metodología:**

Se realizó la traducción y adaptación cultural del cuestionario y se incluyó dentro de una batería de escalas, cumplimentada por 526 auxiliares de enfermería de centros residenciales para personas mayores del Principado de Asturias. Se analizó la validación de la escala a través de la sucesión del análisis factorial exploratorio (AFE) y el análisis factorial confirmatorio (AFC). La consistencia interna se estimó con el coeficiente ordinal ω de McDonald, complementándose con el análisis de fiabilidad test-retest por medio del coeficiente de correlación intraclase (ICC). La validez de criterio se estimó a través de la correlación de la puntuación total de la prueba con las medidas de calidad de vida, incertidumbre laboral, demanda psicológica y apoyo social en el trabajo.

**Resultados:**

Los índices de ajuste de AFE y AFC mostraron que se trata de una prueba unidimensional. Los valores de consistencia interna señalaron una fiabilidad muy alta (ω=0,81) y el ICC fue excelente (r=0,95). La validez de criterio mostró una correlación estadísticamente significativa con todos los constructos estudiados, especialmente con la calidad de vida.

**Conclusiones:**

La presente versión española del NMQ presenta unas buenas cualidades psicométricas en la población de personal auxiliar de enfermería por lo que podría ser una herramienta válida y fiable en la evaluación de los trastornos musculoesqueléticos.

## Abstract (en)

**Background:**

The aim of this study is to translate, culturally adapt, and validate a Spanish version of the Nordic Musculoskeletal Questionnaire for a sample of nursing assistant aides.

**Methods:**

The questionnaire was translated and culturally adapted. Next, it was included in a battery of tests that was completed by 526 nursing assistants working in residential care homes in the Principality of Asturias (Spain). To assess its validity, the Exploratory Factor Analysis and the Confirmatory Factor Analysis were used. The internal consistency was estimated with McDonald’s Omega coefficient (ω), complemented by the test-retest reliability analysis through the intraclass correlation coefficient. The validity of the criteria was established by the correlation between total score on the test and quality of life measures, job insecurity and psychological demand, and social support at work.

**Results:**

The Exploratory Factor Analysis and Confirmatory Factor Analysis adjustment indices confirmed it is a unidimensional test. The internal consistency values indicated very high reliability (ω = 0.81). Similarly, the intraclass correlation coefficient showed statistically significant values and an excellent correlation coefficient (r = 0.95). The validity of the criteria showed a statistically significant correlation with all the constructs studied, particularly with quality of life.

**Conclusions:**

This Spanish version of the Nordic Musculoskeletal Questionnaire has good psychometric qualities for a population of nursing aides and therefore may be a valid and reliable tool for assessing musculoskeletal disorders.

## INTRODUCCIÓN

Los trastornos musculoesqueléticos (TME) se definen como un amplio rango de condiciones inflamatorias y degenerativas que afectan a músculos, tendones, ligamentos, articulaciones, nervios periféricos y estructuras de sostén como los discos intervertebrales^1^. Cuando son producidos o agravados por la actividad laboral, se denominan TME relacionados con el trabajo.

De acuerdo con *la International Labour Organization* (ILO), cerca de 160 millones de casos de enfermedades relacionadas con el trabajo se manifiestan en el mundo anualmente. Entre ellas, los TME representan la segunda enfermedad más frecuente, siendo la mayor causa de baja por enfermedad en los países desarrollados^2^.

Los TME relacionados con el trabajo tienen una etiología multifactorial y tradicionalmente se han asociado a factores de riesgo laborales físicos y ergonómicos como posturas incómodas, movimientos repetidos o manipulación de cargas. Pero hace décadas que se estudia el impacto de los factores de riesgo psicosocial en estas patologías^3^^,^^4^, definidos como *aquellos aspectos del diseño, la organización y la gestión del trabajo, así como del contexto social y ambiental donde tiene lugar, que potencialmente pueden causar daños psicológicos, sociales o físicos*^5^.

Para explicar el vínculo entre estos factores y la salud se han desarrollado diferentes modelos, entre ellos el de Karasek y Theorell^6^ que concibe el estrés laboral como un equilibrio entre las demandas psicológicas del trabajo y el nivel de control de la persona trabajadora sobre ellas. Así, la coexistencia de una alta demanda y un bajo control aumenta el riesgo de estrés, con su consecuente efecto sobre la salud. El apoyo social por parte del personal (compañeros de trabajo) y de las personas al mando o supervisoras puede actuar como elemento moderador que minimiza el riesgo. La relación entre estos factores de riesgo y la presencia de TME ha sido ampliamente puesta de manifiesto en la literatura científica^7^^,^^8^.

Existen riesgos psicosociales emergentes vinculados al contexto del mercado laboral actual. La incertidumbre laboral (IL) es uno de los que ha cobrado mayor interés en el mundo de la investigación en la última década debido a su impacto sobre la salud física y mental. La IL se ha definido como la anticipación subjetiva individual de la pérdida involuntaria del trabajo^9^. La actual flexibilidad contractual, junto a un mercado laboral inestable, aumentan el riesgo de experimentar IL, de modo que se presenta como una medida de la precariedad laboral^10^.

Se puede considerar que ciertos grupos ocupacionales tienen mayor riesgo de desarrollar TME. El personal sanitario, como el personal auxiliar de enfermería que presta cuidados a mayores (compuesto mayoritariamente por personal Técnico en Atención a Personas en Situación de Dependencia, en Atención Sociosanitaria o en Cuidados Auxiliares de Enfermería), frecuentemente está expuesto en el trabajo a riesgos psicosociales que se caracterizan por considerables demandas psicológicas y emocionales y altos niveles de estrés percibido. Estos riesgos, sumados a los factores físicos y ergonómicos ya mencionados, convierten a los TME en una patología habitual dentro de este colectivo^11^^,^^12^.

Ante un problema de salud ocupacional tan prevalente, se han desarrollado diferentes cuestionarios específicos para medir las diferentes dimensiones de esta patología. El cuestionario autoadministrado *Nordic Musculoskeletal Questionnaire* (NMQ) fue diseñado por Ilkka Kuorinka y col^13^ en 1987 para detectar TME relacionados con el puesto de trabajo y evaluar su evolución. Ha sido reconocido y validado internacionalmente^14^^-^^26^. Aunque no permite valorar las causas, es una herramienta válida, fiable y accesible que ha sido ampliamente utilizada en todo tipo de estudios sobre la salud musculoesquelética relacionada con el trabajo^27^, como asociación entre TME y factores de riesgo psicosocial^28^, incluyendo riesgos psicosociales emergentes como la IL^29^^,^^30^, o como impacto de los TME en la calidad de vida relacionada con la salud^31^. A pesar de que el NMQ ha sido traducido, adaptado y validado en diferentes países^14^^-^^25^, solo existe una adaptación en España^26^, específicamente validada en músicos.

El objetivo principal de este estudio es realizar la traducción, adaptación cultural y validación del NMQ en un grupo ocupacional especialmente afectado por los TME: el personal auxiliar de enfermería^11^. El objetivo secundario es validar un método de codificación que permita obtener valores cuantitativos a partir de los resultados dicotómicos del cuestionario con la finalidad de obtener más información a partir de un análisis estadístico más amplio.

## MATERIAL Y MÉTODOS

Este estudio transversal se realizó en los 35 centros de Establecimientos Residenciales para personas mayores de Asturias (ERA), organismo público adscrito a la Consejería de Derechos Sociales y Bienestar del Gobierno del Principado de Asturias (España), entre noviembre de 2021 y junio de 2022. La población diana fue el colectivo de personal auxiliar de enfermería en activo.

Se calculó el tamaño muestral para estimar una proporción del 50%, con una precisión del 5%, en una población finita de 1.237 auxiliares de enfermería del ERA en activo, obteniéndose un tamaño de muestra de 293 auxiliares.

### Procedimiento

Se contactó con la dirección de cada centro para concertar una cita presencial, para enviarles un documento escrito explicativo del proyecto para ser compartido con el personal auxiliar de enfermería, y para proponer horarios de descanso o de menor carga laboral (por ejemplo, a media mañana) de cara a una breve charla presencial, a fin de que asistiera el mayor número de trabajadores posible.

Se impartió la charla presencialmente en cada centro; se explicó el objetivo del estudio y el contenido de los cuestionarios, se entregó un número de cuestionarios que incluyera a todo el personal auxiliar de enfermería en activo del centro (de todos los turnos) y, por último, se les solicitó que trasladasen a sus compañeras la información, tratando de motivar su participación. Se realizó también un vídeo breve con el objetivo de compartir la información de manera multimedia a través de los grupos de *WhatsApp* cuando fuera posible. Durante el proceso de recogida de datos se observó una mayor participación cuando se implicaba al colectivo de auxiliares de enfermería en la entrega y divulgación de la información entre compañeras, y no solamente a la directiva del centro. Por ello, siempre se trató de obtener un enlace de contacto que sirviera de intermediario con las trabajadoras.

El instrumento de investigación, constituido por la batería de cuestionarios autoadministrados que se describirá a continuación, se rellenó en papel con lápiz. El apartado sociodemográfico de la escala recogió información general sobre la persona encuestada: edad (años), sexo (mujer, hombre), y tipo de contrato (fijo, temporal, otro). La batería de cuestionarios también recogió información sobre riesgos ergonómicos mediante el cuestionario de carga física en el trabajo desarrollado por Hollman y col^32^ (que no se incluye en este artículo).

La batería de cuestionarios incluyó una última página en la que los sujetos interesados en participar en futuras fases de la investigación (re-test) debían incluir su teléfono, que también escribirían en la primera página del cuestionario del re-test. De esta manera se codificó a cada participante con los últimos cuatro dígitos de su número de teléfono para poder incluir en la base de datos la información sobre las dos respuestas. En la charla previa se explicó detalladamente el objetivo de esta segunda fase de re-test y la implicación meramente estadística de los datos que se solicitaban para la misma, que no suponían de ninguna manera la pérdida del anonimato en la investigación, como detalla la Ley de protección de datos incluida en el consentimiento informado de los cuestionarios.

Se repitió el procedimiento en cada centro de la ERA: se entregaron los cuestionarios y se volvieron a recoger a los 8-10 días, dejando en este momento los cuestionarios para las personas que accedían a participar en el re-test (lapso de tiempo que sigue las recomendaciones de José María Ramada-Rodilla y col para este tipo de prueba)^33^.

### Instrumentos de medida

- NMQ. *Nordic Musculoskeletal Questionnaire* de Ilkka Kuorinca y col desarrollado con el apoyo del *Nordic Council of Ministers*^13^, muestra buenos parámetros psicométricos con índices de fiabilidad test-retest en tres submuestras de trabajadores que mostraban una concordancia del 77%, así como un coeficiente de correlación con las historias clínicas de las personas participantes que oscilaba entre 0,8 y 1. Consta de una primera sección general que recoge información de nueve partes del cuerpo (cuello, hombros, codos, muñecas/manos, columna dorsal, columna lumbar, caderas/muslos, rodillas y tobillos/pies), en cada una de las cuales se registra de manera dicotómica (sí/no) la presencia de síntomas en los últimos 12 meses, síntomas en los últimos 7 días, y discapacidad producida por los síntomas. La segunda sección recoge información más específica sobre la zona lumbar, el cuello y los hombros, incluyendo información complementaria sobre diferentes variables: sexo, edad, peso, tiempo realizando el trabajo actual, horas de trabajo a la semana, peso, talla y mano dominante. El cuestionario incluye ilustraciones de las partes del cuerpo para facilitar su identificación a las personas que lo cumplimenten.
- EQ5DL. *EuroQoL 5D-5L:* instrumento genérico de medición de la calidad de vida relacionada con la salud que puede utilizarse en población general; se utilizó el índice destinado a población española (https://www.euroqol.org)^34^. Este cuestionario ha mostrado buenas propiedades psicométricas^35^, con una consistencia interna α= 0,75. Cada participante valora su estado de salud, primero en cinco niveles de gravedad sobre cinco dimensiones de la salud: movilidad, cuidado personal, actividades cotidianas, dolor/malestar y ansiedad/depresión. Las puntuaciones oscilan entre -0,654 y 1; a mayor puntuación, mejor estado de salud. Seguidamente debe marcar el punto de la línea vertical milimetrada que va desde 0 (peor estado de salud imaginable) a 100 (mejor estado de salud imaginable) que mejor refleje su valoración del estado de salud global.
- JCQ. *Job Content Questionnaire:* cuestionario que mide los factores de riesgo psicosocial a partir del modelo demanda-control-apoyo de Karasek y Theorell^6^. La validación española realizada en población sanitaria^36^ está compuesta por 22 ítems agrupados en tres dimensiones: demandas psicológicas del trabajo (6 ítems), control sobre el trabajo (7 ítems) y apoyo social por parte de compañeros y supervisores (9 ítems), que se responden mediante escalas Likert 1-4; a mayor puntuación, mayor riesgo psicosocial. El cuestionario presenta una consistencia interna α >0,7. En el análisis de este estudio se han incluido las subescalas de demandas psicológicas del trabajo y apoyo social en el trabajo.
- JIS-8. *Job Insecurity Scale-8:* estudia la dimensión cognitiva y afectiva de la IL^37^, mediante ocho ítems con respuesta tipo Likert 1-5: los cuatro primeros corresponden a la dimensión cognitiva (probabilidad real de perder el empleo) y los cuatro restantes a la dimensión afectiva (miedo a perder el empleo). A mayor puntuación en la escala, mayor percepción de IL. La prueba ha sido validada en España^38^, con una fiabilidad α= 0,88.

Todas las personas participantes en el estudio fueron previamente informadas sobre el tratamiento de los datos que aportaban de acuerdo con la legislación europea para la protección de datos de carácter personal (Ley Orgánica 3/2018, España), y de los objetivos que perseguía esta investigación. Si bien las encuestas anónimas no requieren del consentimiento específico de los participantes, dado que es implícito a la participación en ellas, el consentimiento informado de los participantes se obtuvo a través de la marcación de una casilla incluida para este fin en la batería de cuestionarios.

El estudio llevado a cabo contó con el visto bueno del Comité de Ética de la Investigación del Principado de Asturias (CEImPA 2021.360), así como con la autorización de la dirección del Organismo Autónomo de Establecimientos Residenciales para Ancianos de Asturias (ERA), administración adscrita a la Consejería de Derechos Sociales y Bienestar del Gobierno del Principado de Asturias (España).

### Traducción y adaptación transcultural del NMQ

Este estudio se planteó la traducción, adaptación transcultural y validación al contexto español de la primera parte general del cuestionario NMQ partiendo de la versión en inglés validada por Dickinson y col^39^. Estos autores introdujeron modificaciones tanto en el formato como en la redacción del cuestionario con el objetivo de facilitar su cumplimentación, validando esta versión mejorada del NMQ con una muestra de diferentes grupos ocupacionales. Se ha utilizado y puesto a prueba la codificación propuesta por Fernanda Amaral Pinheiro y col^15^, que genera una puntuación Likert 0-4 para cada ítem (0= ausencia total de síntomas; 1= presencia de síntomas en los últimos 12 meses; 2= síntomas en los últimos 12 meses y sin presencia de discapacidad los últimos 7 días; 3= síntomas en los últimos 12 meses y discapacidad; 4= síntomas en los últimos 12 meses y los últimos 7 días, y discapacidad por estos síntomas). La puntuación total del cuestionario está constituida por la suma de la puntuación de los 9 ítems, oscilando entre 0 (ausencia total de síntomas) y 36 (máxima discapacidad).

Antes de comenzar el proceso de traducción se comprobó que el NMQ es público y puede usarse libremente, por lo que no es necesario obtener permisos legales para su utilización. Se siguieron los cinco pasos determinados por Ramada-Rodilla y col^33^ en esta primera etapa de traducción y adaptación transcultural:

1. *Traducción directa:* Se realizó con la participación de dos traductoras bilingües independientes cuya lengua materna era el castellano; una de ellas es traductora profesional, dedicándose al ámbito científico, y la otra es profesora de español residente en Inglaterra.
2. *Síntesis de traducciones:* El cuestionario es de contenido sencillo y no presentó mayores dificultades en la traducción. Se realizó una síntesis consensuando las discrepancias que pudiera haber entre las versiones de las dos traductoras (Anexo I).
3. *Traducción inversa:* La versión obtenida en castellano fue retrotraducida al inglés por dos traductores bilingües de manera independiente y ciega con respecto a la versión original, confirmando los autores del estudio que entre el cuestionario original y la versión traducida no se detectaban diferencias semánticas o conceptuales que pudieran alterar la comprensión del contenido inicial.
4. *Consolidación por un comité de expertos:* Se constituyó un comité multidisciplinar formado por dos fisioterapeutas, dos psicólogos y un sociólogo que analizaron el contenido de la versión final, confirmando que su lectura era comprensible, sin incluir términos o conceptos dudosos, y análoga al cuestionario original. Los autores del estudio añadieron modificaciones semánticas, como la inclusión de otra categoría en las variables sexo y dominancia de la mano.
5. *Pre-test:* Con el objetivo de analizar la aplicabilidad o viabilidad del cuestionario, se llevó a cabo un pre-test con una muestra heterogénea de 31 participantes de ambos sexos, con rango de edad de 40 a 73 años, diferentes niveles educativos, tanto personal de los sectores público y privado en activo de diferentes grupos ocupacionales (Técnico en Atención a Personas en Situación de Dependencia, Técnico en Atención Sociosanitaria, Técnico en Cuidados Auxiliares de Enfermería,) como personas jubiladas. El cuestionario se acompañó de una hoja donde los sujetos podían registrar las dificultades encontradas a la hora de su cumplimentación.

Finalmente, se elaboró el documento final (Anexo II) teniendo en cuenta aspectos formales del documento para garantizar la accesibilidad e inclusividad a partir de las consideraciones propuestas por la Coordinación Nacional de Accesibilidad^40^, que en este caso consistieron en alinear el formato a la izquierda y utilizar un interlineado mínimo de 1,15, además de utilizar un lenguaje inclusivo en la redacción del texto. Se elaboraron imágenes propias a partir de los diseños originales del artículo de Ilkka Kuorinca y col^13^ para mejorar su nitidez y calidad.

### Análisis estadístico

Las variables categóricas se describieron mediante frecuencias absolutas y porcentajes, y las cuantitativas mediante media y desviación estándar (DE).

En primer lugar, se llevó a cabo un análisis de validación para el cuestionario NMQ a través del análisis factorial exploratorio (AFE) y del análisis factorial confirmatorio (AFC). Para ello, la muestra se dividió aleatoriamente en dos mitades, n= 279 para el AFE y n= 248 para el AFC. Con la muestra completa se analizó tanto la consistencia interna de la escala como la validez de criterio.

Dada la naturaleza ordinal de los ítems del cuestionario y haber observado un valor de asimetría de 12,06 (>1) y correlaciones inter-ítem >0,50 se recomienda aplicar el AFE de correlaciones policóricas^41^, desarrollado con el procedimiento de mínimos cuadrados no ponderados (*unweighted least squares*, ULS) del método paralelo de análisis^42^. Se estudió la adecuación de los datos al AFE mediante la medida Kaiser-Meyer-Olkin (KMO) y la prueba de esfericidad de Bartlett (existe adecuación si KMO >0,75 y Barlettt p <0,05^41^). Se valoró la eliminación de ítems a través de la medida de adecuación del muestreo MSA (*Measure of Sampling Adequacy*, MSA), así como la dimensionalidad con un sistema cruzado de varios indicadores: La media de las cargas absolutas residuales del item (*Mean of Item Residual Absolute Loadings*, MIREAL), con su correspondiente intervalo de confianza al 95% (IC95%); los valores de congruencia unidimensional (UniCo), y el porcentaje de varianza explicada. Se asume unidimensionalidad con MIREAL significativo^43^, valores próximos al 50% del porcentaje de varianza explicada^42^ y UniCo >0,95^41^. El AFE se realizó con el programa FACTOR 12.01.02 desarrollado por la Universidad *Rovira i Virgili*.

De acuerdo con los resultados del AFE, se puso a prueba la estructura factorial de la escala a través del AFC en la segunda mitad de la muestra. Se seleccionó como método de extracción de factores ULS, ya que se adecuado para ítems ordinales^44^ y en muestras de más de 200 casos^45^. El ajuste del modelo fue estimado con los indicadores de residuo cuadrático medio estandarizado (*Standardized Root Mean Square Residual*, SRMR), el error cuadrático medio de aproximación (*Root Mean Square Error of Approximation*, RMSEA), y los índices Tucker-Lewis (TLI), de bondad de ajuste (*goodness of fit index,* GFI) y de ajuste comparativo *(Comparative Fit Index,* CFI), considerando existencia de ajuste de acuerdo a los siguientes criterios: SRMR ≤0,08, RMSEA ≤0,08, TLI >0,90 y CFI ≥0,95^46^^,^^47^. El AFC se desarrolló con la versión 0.16.3 del software JASP, que también fue utilizado en el cálculo de la invarianza factorial.

Se realizó una invarianza factorial de la escala con dos grupos obtenidos de dividir la muestra en función de una variable dicotómica (habitualmente el sexo, pero en este caso el tipo de contrato, considerado como una manera de medir la precariedad laboral^10^), y realizando un análisis de ajuste de modelo para comprobar si la escala se comporta de igual manera en ambos grupos (no existe invarianza, que sería lo adecuado): personal con contrato indefinido y personal con contrato temporal. Para realizar el análisis, se llevaron a cabo cuatro modelos de invarianza^48^: configural, métrica, escalar y estricta, que van del modelo más débil y permisivo (configural) al más fuerte y estricto (estricta), y se van comparando con el modelo base. Se observó cómo cambian los indicadores y en particular cómo cambiaba el CFI (criterio ΔCFI ≤0,01)^49^ así como los valores de ajuste RMSEA (criterio RMSEA ≤0,10) y TLI (criterio TLI >0,90)^46^.

La consistencia interna de la escala NMQ se estimó con los coeficientes omega de McDonald (ω) y alfa de Cronbach (α); valores superiores a 0,07 muestran una consistencia interna adecuada^50^. Esta información se complementó con el análisis de fiabilidad test-retest mediante el coeficiente de correlación intraclase (ICC) estudiado con factores de efectos mixtos entre las pruebas test-retest, asumiendo que valores entre 0,50 y 0,75 son aceptables, entre 0,75 y 0,90 buenos, y mayores que 0,90 excelentes^51^.

La validez de criterio de la Escala NMQ con la codificación ponderada se estimó a través de la correlación de la puntuación total de la prueba con constructos relacionados con ella en la literatura: calidad de vida^31^ (EQ5DL), incertidumbre laboral^29^^,^^30^ (JIS-8), y control del trabajo en las medidas de demanda psicológica y apoyo social^28^ (JQC). Por último, para analizar el comportamiento de la escala se llevó a cabo una regresión lineal con la puntuación total del NMQ como variable independiente y la de calidad de vida (puntuación con el EQ5DL) como variable dependiente.

Tanto los análisis correlacionales y regresivos, como el ICC, se llevaron a cabo con IBM SPSS 25, considerándose significativo un valor de p <0,05.

## RESULTADOS

La población diana estaba constituida por 1.237 auxiliares, seleccionadas a través de un método de muestreo no probabilístico de conveniencia (ofreciendo la posibilidad de participación a todos los sujetos) y obteniendo una muestra final de 526 participantes (tasa de respuesta 42,5%). La edad media fue 49,71 años (rango: 22-66), y el 94,1% eran mujeres (Tabla 1).

**Tabla 1:** Información sobre la muestra

	Total	Mujeres	Hombres
n (%)	526	494 (94,1%)	31 (5,9%)
Edad media (DE)	49,71 (9,56)	0 (10,0)	46 (9,0)
Tipo de contrato			
indefinido	224 (42,6%)	214 (43,3%)	10 (32,3%)
temporal	233 (44,4%)	217 (43,9%)	16 (51,6%)
otro	8 (13,0%)	3 (12,8%)	(16,1%)

DE: desviación estándar.

El test KMO indicó una buena adecuación de la escala para el análisis factorial (KMO= 0,814), al igual que el test de esfericidad de Bartlett (χ^2^= 758,3; grados de libertad = 36; p <0,001).

El valor MIREAL obtenido en el AFE de correlaciones policóricas con método de extracción ULS (0,256; IC95%: 0,200-0,275) permitió asumir que se trata de una prueba unidimensional, asunción confirmada por el valor UniCo (0,959; IC95%: 0,951-0,977) y por el 49,92% de varianza explicada por el modelo de un factor; con un hipotético modelo de dos factores el porcentaje de varianza explicada descendía al 13,7%.

Los nueve ítems del factor presentaron un peso factorial superior a 0,3 y midieron el mismo concepto latente (todos mostraron MSA >0,50), por lo que se decidió mantenerlos (Tabla 2).

**Tabla 2:** Análisis factorial exploratorio del *Nordic Musculoskeletal Questionnaire*

Item	Zona	M	DE	Asimetría	Curtosis	r*	Cargas factoriales
1	Cuello	2,04	1,49	0,075	-1,38	0,465	0,621
2	Hombro)	1,86	1,55	0,157	-1,438	0,482	0,615
3	Codo)	0,6	1,24	1,92	2,2	0,305	0,443
4	Muñeca/mano	1,64	1,62	0,42	-1,409	0,448	0,605
5	Columna dorsal	1,42	1,56	0,62	-1,142	0,463	0,658
6	Columna lumbar	2,31	1,57	-0,248	-1,442	0,487	0,682
7	Cadera/muslo	1,22	1,57	0,877	-0,847	0,395	0,517
8	Rodilla	1,12	1,51	0,979	-0,611	0,362	0,493
9	Tobillo/pie	0,72	1,38	1,692	1,236	0,404	0,627

M: media; DE: desviación estándar; *: correlación ítem-test, todas las correlaciones fueron muy significativas (p< 0,01).

A continuación, se puso a prueba el NMQ con un factor a través del AFC para la otra mitad de la muestra. En este caso, todos los índices de ajuste del modelo estudiados fueron adecuados: SRMR= .075; RMSEA= .070; TLI= .946; CFI= .960; GFI=.942 (IC = 0.931; 0.965).

De acuerdo con los índices de ajuste de AFE y AFC podemos asumir que se trata de una prueba unidimensional.

El número de participantes en la prueba re-test fue de 52. Los valores de consistencia interna (ω= 0,81 y α= 0,82) señalan una fiabilidad muy alta para la escala analizada. Asimismo, el ICC fue excelente (r= 0,95; IC95%: 0,914-0,955) y significativo (p <0,01). Estas evidencias indican una alta fiabilidad para esta versión del NMQ.

El test de invarianza mostró que el incremento CFI entre el modelo base y el más robusto (estricta) se ajusta al criterio propuesto (ΔCFI= 0,01), por lo que no existió invarianza en la Escala NMQ entre las personas que trabajan con contrato indefinido y las que lo hacen con contrato temporal. Los valores RMSEA inferiores a 0,08 y de TLI superiores a 0,90 en todos los modelos de invarianza permiteron asumir un buen ajuste de dichos modelos (Tabla 3).

**Tabla 3:** Modelos de invarianza del cuestionario *Nordic Musculoskeletal Questionnaire* para grupos de contrato indefinido y contrato temporal

Modelo	X^2^	gl	RMSEA	TLI	CFI	ΔCFI
Base	56,535	27	0,07	0,946	0,960	
Configural	85,903	53	0,074	0,939	0,955	0,005
Métrica	102,586	61	0,078	0,933	0,944	0,016
Escalar	124,773	88	0,061	0,959	0,950	0,010
Estricta	124,773	88	0,061	0,959	0,950	0,010

gl: grados de libertad; RMSEA: *Root Mean Square Error of Approximation*; TLI: Tucker-Lewis *Index*; CFI: *Comparative Fix Index*; Δ: incremento.

La validez de criterio mostró una correlación negativa y alta entre la puntuación total del NMQ y la calidad de vida (r= -0,626 con EQ5DL índice y r=-0,657 con la autovaloración del EQ5DL, ambas p <0,001). La puntuación total del NMQ se correlacionó de forma estadísticamente significativa con la IL (puntuación JIS-8, r=0,120), con el apoyo social (JCQ apoyo, r=-0,175) y con la demanda psicológica del trabajo (JCQ demanda, r=0,222).

Finalmente, la puntuación total de la Escala NMQ con el método de codificación propuesto predijo la puntuación de calidad de vida EQ5DL de forma significativa (β = -0,626; p <0,001) y negativa (a mayor puntuación en trastornos músculoesqueléticos obtenida en el NMQ, peor calidad de vida), explicando un 39,1% del ajuste del modelo (R2 ajustado).

## DISCUSIÓN

El propósito de este estudio era traducir y adaptar culturalmente el NMQ en español validándolo con una muestra de auxiliares de enfermería, al tratarse de un grupo ocupacional que presenta una alta prevalencia de esta patología.

La muestra analizada está compuesta por un 94,1% de mujeres, proporción algo superior pero congruente con la ocupación en España del sector sanitario y sociosanitario; los datos del Instituto de la Mujer (Gobierno de España) reflejan que un 84% del personal de enfermería, de residencias para mayores y de personas dependientes son mujeres^52^.

En este estudio se ha realizado un análisis exhaustivo de las propiedades psicométricas del NMQ, proponiendo métodos de corrección que permitan realizar análisis estadísticos más completos con la información obtenida. El tamaño muestral utilizado (n= 526) es el mayor de las validaciones del NMQ publicadas hasta el momento, que oscilan entre los 40^14^ y 312 sujetos^26^. También es similar el grupo ocupacional elegido, perteneciente al sector sanitario al igual que los trabajos de Narges Arsalani y col^17^ y Turhan Kahraman y col^24^.

La traducción se ha realizado utilizando la parte general del cuestionario a partir de la modificación de Dickinson y col^33^, de igual modo que los trabajos realizados en Brasil por de Barros y Alexandre^14^ y Fernanda Amaral Pinheiro y col^15^. Otros estudios^19^^-^^22^^,^^23^ han utilizado la parte general del cuestionario original de Ikkla Kuorinca y col^13^ o su versión extendida (NMQ-E)^23^^,^^25^. Tan sólo Gobba y col^18^ y Rosa Gómez-Rodríguez y col^26^ han realizado una traducción y validación del NMQ completo.

Como novedad, esta es la única validación de las existentes que analiza la estructura de la prueba a través de AFE y de AFC. Los resultados favorables alcanzados mediante este procedimiento aportan una validez estadística inédita hasta ahora para la prueba, reflejando la calidad del instrumento. A partir de estos análisis se evidencia también la viabilidad del método de codificación realizado previamente por Fernanda Amaral Pinheiro y col^15^ como corrección para tratar las variables dicotómicas como continuas, abriendo nuevas posibilidades de análisis en los estudios epidemiológicos. También incorpora como novedad el estudio de la invarianza factorial de la escala que, no habiéndose realizado en ninguna de las validaciones previas del NMQ, constituye una prueba añadida de las buenas cualidades psicométricas del cuestionario. En este caso se realizó el análisis de invarianza dividiendo los grupos en función del tipo de contrato (indefinido o temporal), una variable que la literatura previa ha propuesto como medida de precariedad y consecuente factor de riesgo psicosocial vinculado a los TME^10^^,^^29^^,^^30^. El resultado refleja que se trata de una medida robusta para el fenómeno que permite observar.

En cuanto a la consistencia interna, se optó por calcular también el estadístico omega de McDonald, a diferencia de la mayoría de las validaciones previas que utilizan el alfa de Cronbach^16^^,^^18^^,^^20^^,^^23^ o el coeficiente de fiabilidad de Kurder-Richardson^17^^,^^22^^,^^26^. El resultado de este trabajo es similar al resto de estudios, que muestran una consistencia interna buena-alta, lo que indica que los ítems del cuestionario se correlacionan entre sí de manera adecuada para medir el constructo que evalúan.

El ICC obtenido al evaluar la fiabilidad test-retest mostró una correlación excelente y superior a la de los estudios de Narges Arsalani y col^17^ (r=0,78) y Nuray Alaca y col^23^ (r=0,88). La mayoría de las validaciones previas han evaluado la fiabilidad test-retest utilizando el coeficiente *Kappa* de Cohen con resultados igualmente buenos-excelentes.

Las validaciones anteriores del NQM utilizaron muestras clínicas y de pequeño tamaño^15^^,^^19^^,^^20^^,^^25^, mientras que la muestra utilizada en este estudio es de mayor tamaño y está formada por profesionales en activo. Ello aporta mayor certeza estadística para el uso del NQM al tiempo que extiende su uso a contextos de prevención de riesgos laborales y de investigación. El análisis de validez presentado siguió el procedimiento de otros estudios similares^53^^,^^54^: se evaluó la validez de criterio a partir de la correlación de la puntuación total obtenida a partir de la codificación de la escala NMQ propuesta en este estudio con variables previamente relacionadas con la presencia de TME en la literatura científica (calidad de vida^31^, incertidumbre laboral^29^^,^^30^, demanda psicológica del trabajo y apoyo social en el mismo^28^), obteniéndose correlaciones estadísticamente significativas con los constructos estudiados, especialmente con la calidad de vida. Además, la puntuación del NMQ mostró un ajuste moderado para predecir la puntuación de calidad de vida EQ5DL. Todo ello aporta evidencia sobre la validez de la prueba en la muestra española a la que está destinada.

Como principal limitación de este estudio surge su diseño transversal, que no permite asumir causalidad, solo asociación. La validez externa de los resultados obtenidos puede no estar asegurada para hombres (escasa representación masculina en la muestra estudiada) ni para otros estamentos profesionales ni mismos estamentos con otro tipo de labores. Por ello, sería conveniente realizar más trabajos académicos que estudiaran su validez y estructura con otros grupos profesionales del ámbito sanitario, desarrollando futuras líneas de investigación que incluyan esta herramienta en el estudio de los TMERT.

La principal conclusión de este estudio es que la versión traducida y validada del NMQ que se presenta, constituye una herramienta de investigación apropiada para el estudio de los TME relacionados con el trabajo desempeñado por personal auxiliar de enfermería o personal sanitario con funciones similares en España. La metodología utilizada aplicó un proceso estadístico riguroso para confirmar las cualidades psicométricas del cuestionario, respaldando los resultados de la evidencia científica previa sobre su fiabilidad y validez, pero también aporta información relevante que no se había analizado previamente, como el análisis factorial exploratorio y la invarianza factorial de la escala. Además, se valida un método de codificación que permite tratar las variables dicotómicas como continuas, aumentando las posibilidades de estudio que ofrece el cuestionario.

## ANEXO I. Memoria de la síntesis de traducciones

**Table:**

Original	Traducción	Modificaciones
*The date of inquiry*	Fecha de cumplimentación	Se cambió el orden a día, mes, año.
		Se cambió *consulta* por *cumplimentación*, ya que se considera más adecuado para cuestionarios.
*Sex*		Se añadió *otro* como opción de respuesta.
*How tall are you*	Cuál es su estatura	Se cambió *cuánto mide* por *cuál es su estatura.*
*Are you right-handed or left-handed*	Se considera diestro o zurdo	Se cambió *es usted diestro o zurdo* por *se considera diestro o zurdo*.
		Se añadió ambidiestro como opción de respuesta.
*Locomotive organs*	Aparato locomotor	
*You may be in doubt as to how to answer, but please do your best anyway*	Es posible que te surja alguna duda mientras tratas de rellenar el cuestionario; por favor, intenta responder lo que refleje mejor tu caso	Se eliminó el párrafo: *por favor, no se olvide de contestar a todas las preguntas, incluso aunque nunca haya tenido problemas en esas partes de su cuerpo*, porque creaba confusión en la tabla de preguntas, ya que las dos columnas de la derecha especifican que solo se contesten si se respondió afirmativamente en la primera columna.
*Neck*	Cuello	
*Low back*	Columna lumbar	
*Upper back*	Columna dorsal	
*Ache, pain or discomfort*	Dolor molestias o incomodidad	Previamente se barajó *dolor, fatiga o incomodidad*
